# Experience in the treatment of long-gap esophageal atresia by intraluminal esophageal stretching elongation

**DOI:** 10.3389/fped.2024.1367935

**Published:** 2024-03-08

**Authors:** Ning Zhang, Wenjie Wu, Yujia Zhuang, Weipeng Wang, Weihua Pan, Jun Wang

**Affiliations:** ^1^ Department of Pediatric Surgery, Children’s Hospital of Soochow University, Suzhou, Jiangsu, China; ^2^ Department of Pediatric Surgery, The Affiliated Xuzhou Children’s Hospital of Xuzhou Medical University, Xuzhou, Jiangsu, China; ^3^ Department of Pediatric Surgery, Xinhua Hospital Affiliated to Shanghai Jiao Tong University School of Medicine, Shanghai, China

**Keywords:** esophageal atresia, long-gap, intraluminal esophageal stretching, elongation, postoperative complications

## Abstract

**Objective:**

To summarize the experience with intraluminal esophageal stretching elongation (ILESE) in the successful treatment of long-gap esophageal atresia (LGEA) at a single center.

**Methods:**

Clinical data of 68 neonates who underwent LGEA between February 2015 and January 2022 were retrospectively analyzed. Four patients died of multiple associated severe malformations and did not undergo ILESE. Esophageal anastomosis was successfully performed in 60 cases (93.75%) and failed in 4 cases (6.25%) treated with ILESE. The ILESE techniques, esophageal reconstruction, results, postoperative complications, and follow-up treatment were analyzed.

**Results:**

The beginning time of performing ILESE preoperation was 53.4 ± 39.4 days after birth, and the age of esophageal reconstruction was 122.2 ± 70.3 days after birth in 60 cases. The gap length of proximal and distal esophageal segments which were evaluated the first time at admission was 4.8 ± 1.3 vertebral bodies, whereas the gap before anastomosis was −0.46 ± 0.90 vertebral bodies. Among the patients with esophageal primary-anastomosis, 55 received thoracoscopic surgery, and 5 underwent thoracotomy in the early stage. Of the 60 children with ILESE, 58 underwent end-to-end esophagostomy, of which 17 cases were combined with circular esophagotomy (livaditis), and 2 cases of esophageal lengthening were combined with the reversal of the ligulate loop of the proximal esophagus (flap). Overall, 59 cases were cured (98.3%), and 1 patient died of respiratory failure postoperatively. All patients were followed up for 7–96 months. Postoperative anastomotic leakage occurred in 16 patients (27.6%), all of whom were successfully treated conservatively. Anastomotic stenosis occurred in 49 cases (83.1%), all of which were successfully managed by non-surgical treatment, including 12.7 ± 9.3 times of esophageal balloon dilatation and 2 cases of stent dilatation. Gastroesophageal reflux occurred in 44 patients (74.6%), including associated or acquired esophageal hiatal hernia in 22 patients, and Nissen fundoplication was performed in 17 patients.

**Conclusions:**

ILESE is an effective method for prolonging the proximal and distal esophagus of the LGEA to reconstruct esophageal continuity using its esophageal tissue, with an efficacy rate of 93.75%. Postoperative anastomotic stricture and gastroesophageal reflux are common and require long-term, standardized follow-up and treatment.

## 1 Introduction

In 2017, the International Esophageal Atresia Network Association defined long-gap esophageal atresia (LGEA) as esophageal atresia (EA) of the abdominal airless, including type I and type II, and clarified that LGEA was more complex than EA with a distal tracheoesophageal fistula (1). In 1974, Meyers reported that the natural esophagus should be greatly protected because there is no other tube to replace its function of conveying food from the mouth to the stomach (2). Currently, the prevailing view of pediatric surgery academia worldwide is to reconstruct esophageal continuity using primary-anastomosis tissue (1, 3). Different techniques are used for esophageal lengthening, such as magnetic anastomosis and thoracoscopic internal traction techniques (4, 5). Mechanical extension, such as the Foker and Kimura techniques, is effective in determining the length of the esophagus, and intraluminal esophageal stretching elongation (ILESE) is one such technique. Howard first reported delayed anastomosis of the esophagus in the treatment of LGEA by bougienage stretching of the proximal segment (6), although few follow-up reports have indicated that it may be related to long-term gastrostomy after surgery and related complications caused by proximal esophageal secretions. However, these issues have been resolved through improvements in perioperative management. In 2018, our center reported the successful treatment of 12 LGEA cases with the ILESE technique for the first time in China (7), and the technique was further applied and improved during follow-up. This study reviewed and analyzed patients in whom the ILESE technique was applied before esophageal anastomosis and summarized relevant experiences for clinicians who may select the ILESE technique for LGEA treatment.

## 2 Materials and methods

### 2.1 Materials

In total, 68 neonates with LGEA were either admitted or transferred to the Department of Pediatric Surgery, Xinhua Hospital Affiliated to Shanghai Jiao Tong University School of Medicine between February 2015 and January 2022, including 63 type I and 5 type II cases. The electronic hospitalization data and outpatient follow-up data of children in the group were collected. This study was approved by the Medical Ethics Committee of Xinhua Hospital affiliated to Shanghai Jiaotong University (XHEC-C-2020-115-1), and the patients' family members signed an informed consent form.

### 2.2 ILESE technique

**Figure 1:**
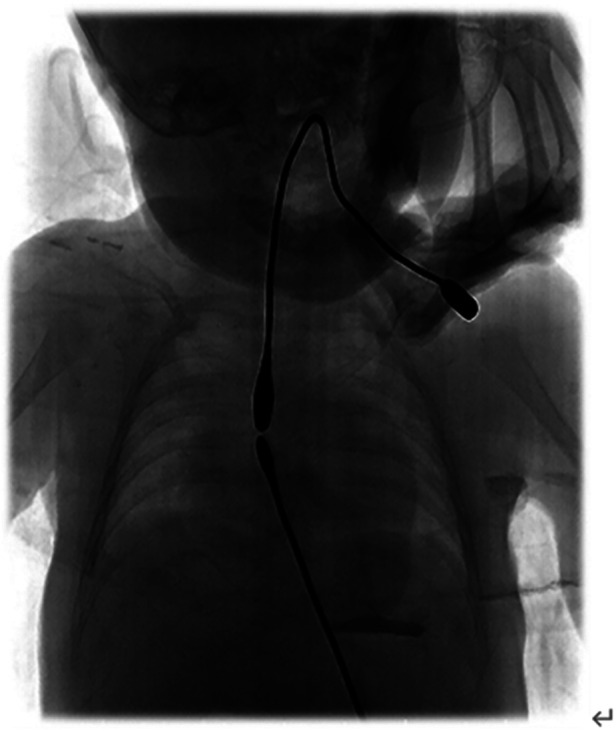
The esophagram showed the gap distance was 0 vertebral body after ILESE.

Patients who underwent gastrostomy in our hospital commonly started ILESE via the proximal and distal esophagus 2 weeks postoperatively. Patients who underwent gastrostomy at the other hospital began to elongate their esophageal segments after being transferred to our hospital. The bougie [size*φ*5–6, B10105, Shanghai Medical Instruments (Group) Ltd., Corp. Surgical Instruments Factory] was inserted into the proximal pouch of the esophagus through the oral cavity, and a downward longitudinal force was applied to the distal esophagus to elongate its length through the combined action of tissue stretching and growth. The bougie was placed into the distal pouch via gastrostomy, and elongation was performed using the same bougie to provide upward pressure simultaneously. The surgeon evaluated the proximal and distal esophageal gaps, shape, and flexibility. Proximal and distal bougienage stretching was performed for 10–15 min in wards but usually by a certain doctor for one certain patient because he can roughly know the direction and how to adjust the angle of the bougie, and esophagography was performed every 2 weeks to evaluate esophageal growth and distance. Under the action of ILESE, proximal and distal esophageal rendezvous (Figure 1), or partially overlapped under radiographic monitoring, thoracotomy, or thoracoscopic end-to-end esophagostomy can be performed.

### 2.3 Operation method

In the left recumbent position, the right upper limb was straightened, raised, and fixed above the head, and the right armpit was fully exposed. Thoracoscopic surgery: A 5 mm Trocar was placed as an observation hole under the right posterior axillary line, and then a 3-mm/5-mm Trocar was placed as an operation hole at the 3/4 and 6/7 ribs of the midaxillary line, and the three points formed a triangle. In thoracotomy, the surgical incision site is located at the intercostal space of the spine, where the proximal and distal esophagus intersect on imaging examination. Thoracoscopic surgery should be performed whenever possible. Its advantage lies in the entire view of the thoracic cavity, which is beneficial for fully dissecting and dissociating the proximal and distal esophagus, avoiding the difficulty of fully dissecting and dissociating the proximal and distal esophagus simultaneously during thoracotomy. Positive 5 atm pressure in the chest compresses the lungs and exposes the posterior mediastinum. A no. 10 gastric tube was inserted into the proximal esophagus to assist in dissociation. Meanwhile, pediatric surgeons should pay attention to separating the esophagus from the trachea and avoiding damage to the trachea, blood vessels, nerves, and surrounding important structures and tissues, and should not cut off the azygos veins without necessity. The tissues of the proximal and distal pouches were cut off, and both ends were sutured intermittently with a 5-0 PDS suture. When the anastomotic tension is too high, a livaditis operation or reversal of the ligulate loop of the proximal esophagus (flap operation) can be considered.

### 2.4 Results of the analysis

The basic data of the neonates, outcomes of ILESE and esophageal reconstruction, and operative complications were collected and analyzed. Patients were followed up via telephone and face-to-face surveys. Continuous variables are expressed as means ± standard deviations. Categorical variables were described as absolute frequencies and percentages. The Statistical Package for Social Sciences (SPSS) version 26 (SPSS Inc., Chicago, Illinois, USA) was used for analysis.

## 3 Results

Among the 68 cases of LGEA, 63 were type I and 5 were type II. All patients were followed up for 7–96 months, monthly for the first three months and 3–6 months after 3 months. Four type I patients who did not undergo ILESE died before surgery because of severe cardiac malformations, such as pulmonary artery stenosis, atrial septal defect, and chromosome abnormalities. 64 cases (59 type I and 5 type II) were enrolled in the group, and 60 patients (56 type I and 4 type II) successfully underwent ILESE and complete primary-anastomosis of the esophagus. One patient died of respiratory failure after surgery postoperatively, with a success rate of 93.75%. In total, 35 cases of gastrostomy were performed at our hospital; ILESE was performed successfully in all cases, and primary-anastomosis reconstruction was completed. Meanwhile, 29 cases of gastrostomy were performed in outer hospitals. Among them, four cases began ILESE at the initial age of 109.5 ± 82.4 days, and the preoperative ILESE time was 148.5 ± 75.5 days. The proximal and distal esophagus could not rendezvous or cross within a vertebral body to complete esophageal anastomosis; thus, four patients underwent colon interposition for esophageal replacement. Three cases of esophageal lengthening in the outer hospital failed to meet the requirements of surgery, and a primary-esophagostomy was successfully performed after transfer to our hospital, followed by the ILESE technique. The general clinical data of patients successfully undergoing ILESE are presented in Table 1.

**Table 1:** Demographics of patients with LGEA.

Clinical data	60 cases
Male	35 (58.3%)
Gestational age (W)	37.4 ± 1.8
Birth weight (g)	2,603.31 ± 530.72
After gastrostomy in the outer hospital	25 (41.7%)
G-tube gastrostomy	15 (25.0%)
Gross type	
Type A	56 (93.3%)
Type B	4 (6.7%)
Concomitant deformity	
Cardiovascular malformation	27 (45.0%)
Anorectal malformation	2 (3.3%)
Urinary system malformation	1 (1.7%)
Skeletal system deformities (including congenital hip dysplasia, scoliosis, rib deformities)	6 (10.0%)
Respiratory malformation	3 (5.0%)

LGEA, long gap esophageal atr.

### 3.1 ILESE and operation outcomes

The initial age of starting ILESE was 68.8 ± 59.0 days after birth. The preoperative ILESE time was 53.4 ± 39.4 days. The age of esophageal reconstruction was 122.2 ± 70.3 days after birth in 60 cases (93.75%) of esophageal auto anastomosis. The gap lengths of proximal and distal esophageal segments which were evaluated for the first time at admission were 4.8 ± 1.3 vertebral bodies, and the gap lengths between esophageal segments were −0.46 ± 0.90 vertebral bodies before anastomosis. Among patients with esophageal primary-anastomosis, 55 received thoracoscopic surgery, and 5 underwent thoracotomy. Esophageal anastomosis was achieved in 19 cases using other lengthening techniques, including circular myotomy (livaditis) in 17 cases, proximal myotomy in 9 cases, and proximal and distal myotomy simultaneously in 8 cases. The diameters of the proximal and distal esophagus were significantly different between the two cases, and a lengthening technique involving the reversal of the ligulate loop of the proximal esophagus (flap) was performed. In three cases, the pouch was perforated when the proximal segment was prolonged, resulting in mediastinal infection, which healed after indwelling thoracic and esophageal drainage and anti-infective treatment, following thoracoscopic esophagostomy after performing ILESE again.

### 3.2 Postoperative complications

Overall, 16 patients had anastomotic leakage, with an incidence of 27.6%, and all of them were cured by active treatment, such as accurate drainage through the leakage, anti-infective treatment, and nutritional support. Anastomotic strictures occurred in 49 patients (83.1%), of which 29 (49.2%) were refractory. All children underwent esophageal balloon dilatation; the number of dilatations was 12.7 ± 9.3 times, and meanwhile, esophageal stent dilatation was performed in 2 two cases. Overall, 44 cases (74.6%) had symptoms such as vomiting, feeding difficulty, and recurrent respiratory infection and were diagnosed with gastroesophageal reflux by upper gastrointestinal radiography and esophageal PH monitoring. Moreover, 22 cases (37.3%) were complicated by esophageal hiatal hernia, of which 17 (28.8%) underwent laparoscopic esophageal hiatal hernia repair and Nissen fundoplication, and reflux symptoms improved. The other five cases (8.5%) were improved by postural feeding and administering the minimum effective dose (0.5 mg/kg/day) of proton pump inhibitors (PPIs), and the symptoms such as vomiting and feeding difficulties were significantly improved and follow-up was observed. Four patients (6.8%) developed tracheoesophageal fistula postoperatively, of whom one patient who underwent the Foker operation in the outer hospital failed. All children underwent tracheoesophageal fistula repair and recovered well postoperatively. The postoperative complications of patients are presented in Table 2.

**Table 2:** Postoperative complications of self-anastomosis of the esophagus after ILESE.

Postoperative complications	59 cases
Anastomotic leakage	16 (27.6%)
Anastomotic stricture	49 (83.1%)
Refractory anastomotic stricture	29 (49.2%)
Gastroesophageal reflux	44 (74.6%)
Esophageal hiatal hernia	22 (37.3%)
Tracheoesophageal fistula	4 (6.8%)

ILESE, intraluminal esophageal stretching elongation.

## 4 Discussion

LGEA is a special type of EA, with types I and II accounting for 7% and 2% of EA, respectively (8). Owing to the long gap between the proximal and distal esophageal pouches, performing primary-anastomosis of the reserved esophagus is impossible immediately after birth, which remains the main challenge faced by pediatric surgeons (9). Shieh has reported that the success rates of primary and secondary repairs of LGEA (once repaired by EA) were 96% and 68%, respectively (10). Of the 64 children enrolled in this study, 63 (98.4%) survived. Among them, 60 were successfully treated with delayed esophageal primary-anastomosis after ILESE, with a success rate of 93.75%.

Various techniques have been used to promote esophageal prolongation and preserve the native esophagus. The easiest way to achieve increased segments seems to be to simply provide the esophagus time for spontaneous growth without invasive manipulations. Puri has reported that the swallowing reflex of neonates and gastroesophageal reflux can stimulate the growth of the proximal and distal esophagus, respectively, with a maximum growth period of 8–12 weeks after birth (11). The anastomosis can be attempted when the distance between the proximal and distal esophagus is within two vertebrae in the natural state. Andrea Conforti et al. reported that 5 LGEA patients were treated by magnamosis and no anastomotic leak was found but all patients developed anastomotic stenosis (4). Mechanical extension is an effective method for determining the length of the esophagus in patients with LGEA. Its principle is to apply a longitudinal traction force to the pouch of the esophagus, and the length of the esophagus is effectively increased through tissue stretching or growth to achieve esophageal primary-anastomosis. Mammoto et al. confirmed that mechanical forces, such as genetic and chemical signals (12). Internal traction (Foker), external traction (Kimura), and various improved traction techniques are based on this principle (13, 14).

van Tuyll van Serooskerken et al. reported that 11/13 patients (85%) were treated by thoracoscopic external traction technique and all patients required multiple dilatations and 10 patients required anti-reflux surgery (5).

Since Rothenberg and Lobe successfully performed the first thoracoscopic repair of esophageal atresia in 1999 (15), minimally invasive surgery has been increasingly used for EA. Thoracoscopic esophageal reconstruction of the LGEA has the advantage of a wide field of vision and is conducive to the full dissociation of the esophagus over a large distance while avoiding complications such as skeletal muscle deformities caused by thoracotomy (16, 17). In the present study, 55 patients underwent successful thoracoscopic reconstruction after ILESE. When dissociating the proximal and distal segments, pediatric surgeons should close the esophageal wall to avoid damage to the vagus nerve, trachea, and other surrounding tissues, and use a knot pusher to tie and secure the knot, which can make the knot firm and not loose. Moreover, the esophageal wall should be fully protected, and the cutting tissue of the proximal and distal esophageal pouches should be used to relieve anastomotic tension and prepare for emergencies.

When anastomosis is difficult or anastomotic tension is high, it can be combined with other lengthening techniques to achieve esophageal anastomosis. In 1973, Livaditis first reported the use of proximal segment myotomy to elongate the length of the esophagus, which can be extended by 0.5–1.0 cm when one or two circular myotomies are performed at the segment of the proximal esophagus (18–20). The blood supply to the proximal segment from the sub-thyroid artery runs along the submucosa (18); therefore, dissociating the proximal segment and performing a circular myotomy is safe (21–23). The supply vessels of the distal esophagus come from the branches of the aorta, intercostal, and pericardial vessels, which are prone to ischemia after dissociation; therefore, cutting the open distal muscular layer is rarely recommended, although some scholars have reported successful incision of the distal muscular layer for esophageal reconstruction (24, 25). The esophageal mucosa at the incision site of 17 patients receiving livaditis surgery was well-protected, and esophageal reconstruction was completed successfully in this study. Brown has reported that the reversal of the ligament loop of the proximal esophagus (flap) can be used as a treatment for LGEA (26). In this group, the distance between the proximal and distal esophagus in two children was still >1 vertebral body after ILESE and a great difference in the diameter of the proximal and distal esophagus was observed. Therefore, flap operation and esophagostomy were performed, and esophagostomy was performed successfully.

Esophageal reconstruction was successfully completed after ILESE in the children who underwent gastrostomy at our hospital. Esophageal lengthening failed in three children with gastrostomy in the outer hospital, one patient underwent the Foker operation to tear the pouch of the esophagus, and two patients failed to undergo ILESE. The perforation of the pouch in three children who underwent ILESE in our hospital may be related to poor flexibility of the esophageal wall and a large longitudinal force. Colon replacement of the esophagus was performed in four children with gastrostomy in other hospitals. To improve the success rate of ILESE and reduce complications, our experience is as follows. First, ILESE should begin as soon as possible when the sinus is formed after gastrostomy. Second, the ILESE technique is best performed in an experienced esophageal atresia diagnosis and treatment center. Third, during the first ILESE, the distance between the proximal and distal esophagus, esophageal shape, and flexibility should be evaluated under dynamic x-ray monitoring. Lastly, ILESE should be performed by the same doctor.

The incidence of anastomotic leakage in this study was 27.1%, which is similar to the 28.7% incidence after delayed anastomosis of the LGEA reported by Friedmacher et al. (27). All anastomotic leakages in this group were conservatively cured, of which three cases had large anastomotic leakage and encapsulated pleural effusion. Cui has reported that thoracic lavage can effectively promote the healing of an anastomotic fistula. However, a long 2.5-cm transverse incision must be made between the second rib of the midline of the clavicle, and the drainage tube must be replaced, which causes secondary trauma to the child (28). Under low-dose x-ray monitoring, placing a drainage tube through the esophageal anastomotic leakage into the thoracic cavity and continuous negative pressure drainage was simple, safe, and effective. Simultaneously, placing negative-pressure drainage near the anastomotic leakage in the esophageal cavity can effectively prevent saliva and reflux from flowing into the thoracic cavity and promote healing. Tracheoesophageal fistula is unrelated to the ILESE technique itself, although it is related to multiple operations, postoperative anastomotic leakage, and high anastomotic tension. One patient in this group was referred to our hospital after the failure of the Foker operation, and anastomotic leakage occurred after second-stage esophageal reconstruction. Anastomotic leakage occurred in three cases after one-stage esophageal reconstruction after ILESE. After the esophagus and trachea were completely separated and the fistula was repaired, air leakage was monitored by intrapleural water injection and airway pressurization to ensure tracheal integrity.

In a systematic review of 57 articles, Stadil reported that the incidence of anastomotic stenosis in the first year after surgery was 61.9% (29). Anastomotic stenosis is defined as stenosis at the level of the esophageal anastomosis found detected on upper gastrointestinal angiography or endoscopy with related symptoms such as dysphagia (30). Although some patients demonstrated narrow anastomotic levels on angiography or endoscopy, no clinical symptoms were observed. For example, small infants do not develop dysphagia when they consume oral milk, and dysphagia occurs only after the addition of complementary foods as they grow older. For such children, we defined it as anastomotic stenosis; therefore, the incidence of anastomotic stenosis was high (83.1%). Additionally, the occurrence of anastomotic stenosis is related to the EA type (31). In our study, all enrolled patients had type I or type II EA, and a correlation was identified between tension anastomosis and partial ischemia. Anastomotic refractory stricture refers to the inability to successfully remediate the anatomic problem to obtain age-appropriate feeding possibilities after a maximum of five dilation sessions with maximal 4-week intervals (32). Patients with refractory strictures after LGEA are related to proximal and distal gaps and the age at esophageal reconstruction. The incidence of refractory strictures in this group was 49.2%, all of which were successfully treated with conservative treatments such as balloon dilatation and esophageal stent implantation.

Postoperative gastroesophageal reflux in patients with LGEA may be related to poor peristaltic function of the esophagus, high anastomotic tension, and nerve injury during esophageal repair (33). Burjonrappa has reported that nine patients with LGEA underwent delayed one-stage anastomosis, eight patients were diagnosed with reflux during gastroscopy, with an incidence of 88.8%, and four patients underwent anti-reflux surgery (34). Sri Paran has reported that 21 patients with LGEA underwent delayed one-stage anastomosis, and 14 patients had symptomatic gastroesophageal reflux (66%); among them, 5 patients received anti-reflux surgery (35). Koivusalo has reported that the proportion of gastroesophageal reflux associated with EA tended to increase over time (36). This study also observed that the symptoms of reflux could be temporarily relieved by adjusting feeding, such as increasing milk consistency or postural treatment, and routine oral PPI (0.5 mg/kg/day) for at least 3 months. However, gastroesophageal reflux is aggravated over time in children with LGEA, and anti-reflux surgery is considered by some pediatric surgeons to be a predictable step (35, 37, 38). In this group, 17 children underwent anti-reflux surgery, and more than half of them received surgical treatment after conservative treatment and follow-up for at least half a year.

The limitations of this study lie in its retrospective design. Moreover, follow-up data are cross-sectional, and longitudinal research data, such as the growth, development, and nutrition of children after discharge, are lacking. Therefore, as a next step, we will conduct a forward-looking, longitudinal data study.

## 5 Conclusion

ILESE is an effective method for prolonging the proximal and distal esophagus of the LGEA to reconstruct esophageal continuity using its esophageal tissue, with an efficacy rate of 93.7%.

## Data Availability

The raw data supporting the conclusions of this article will be made available by the authors, without undue reservation.
